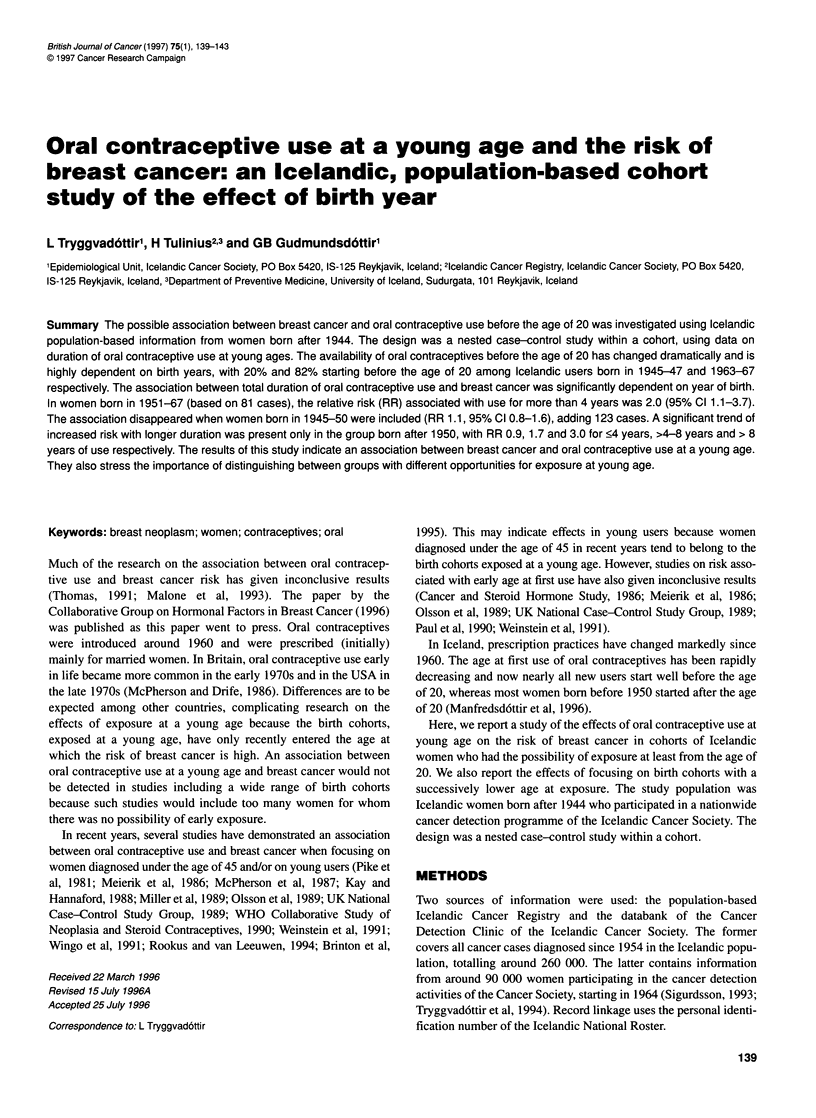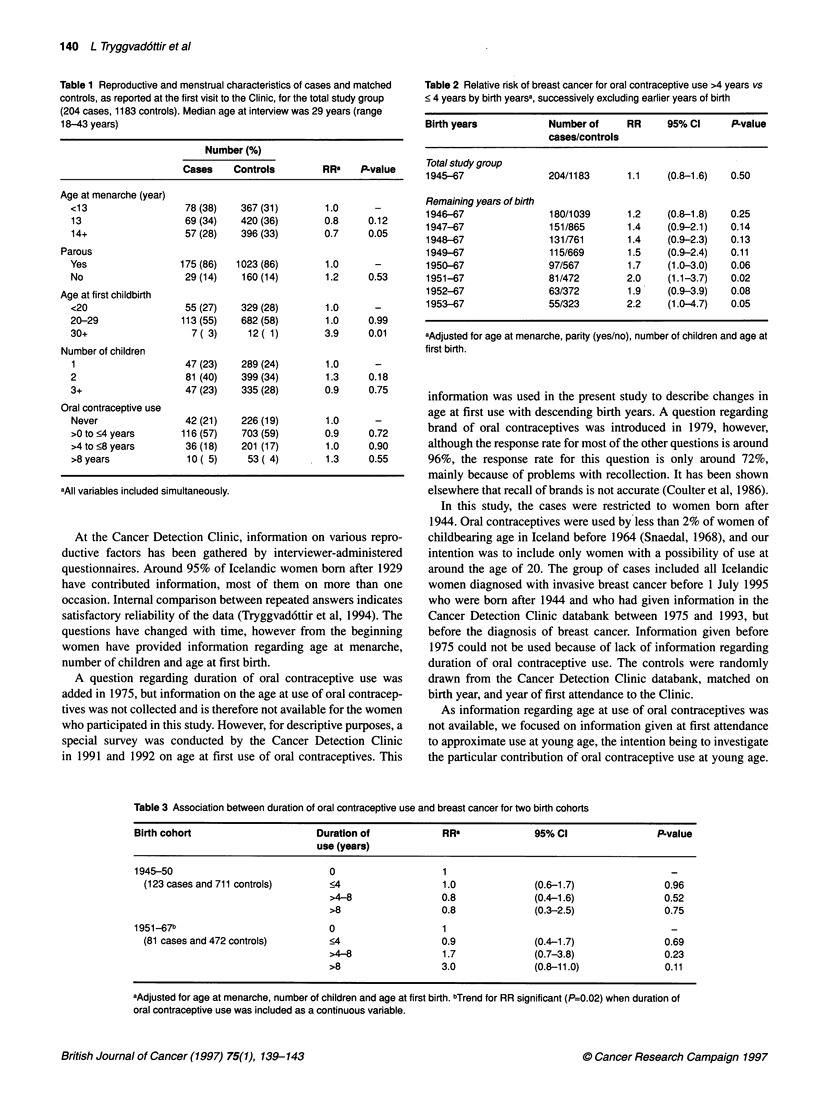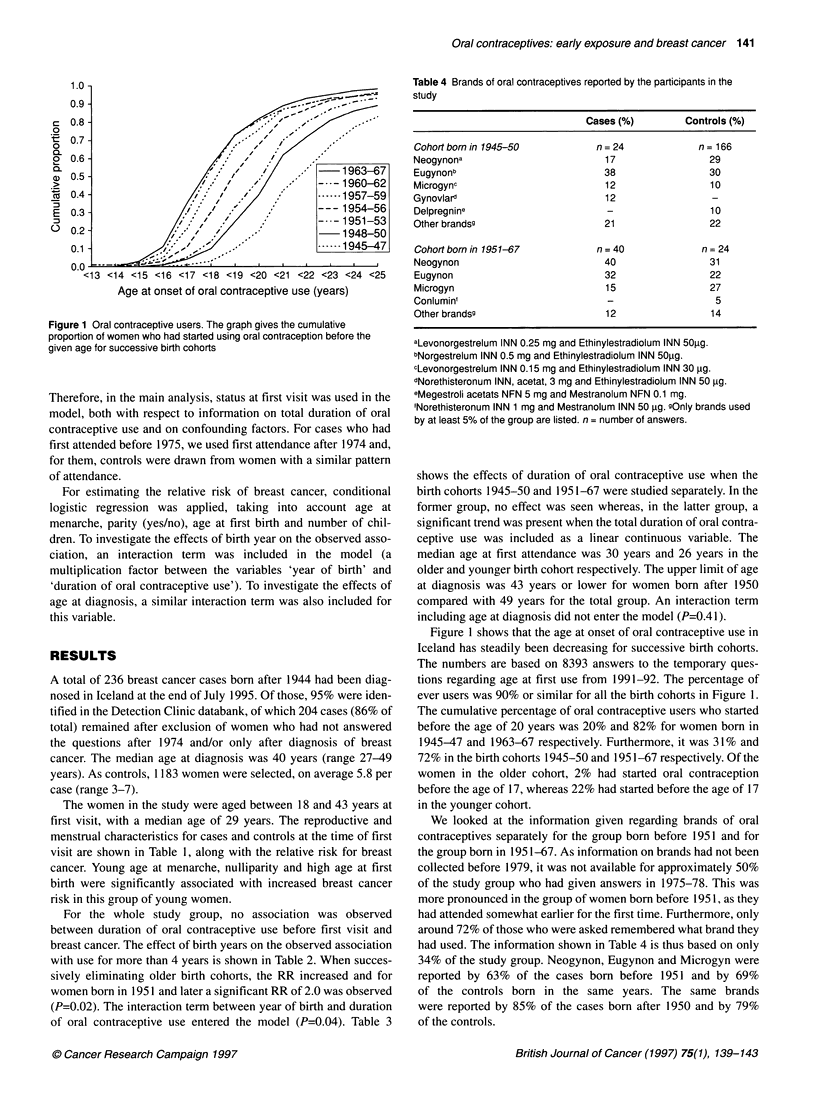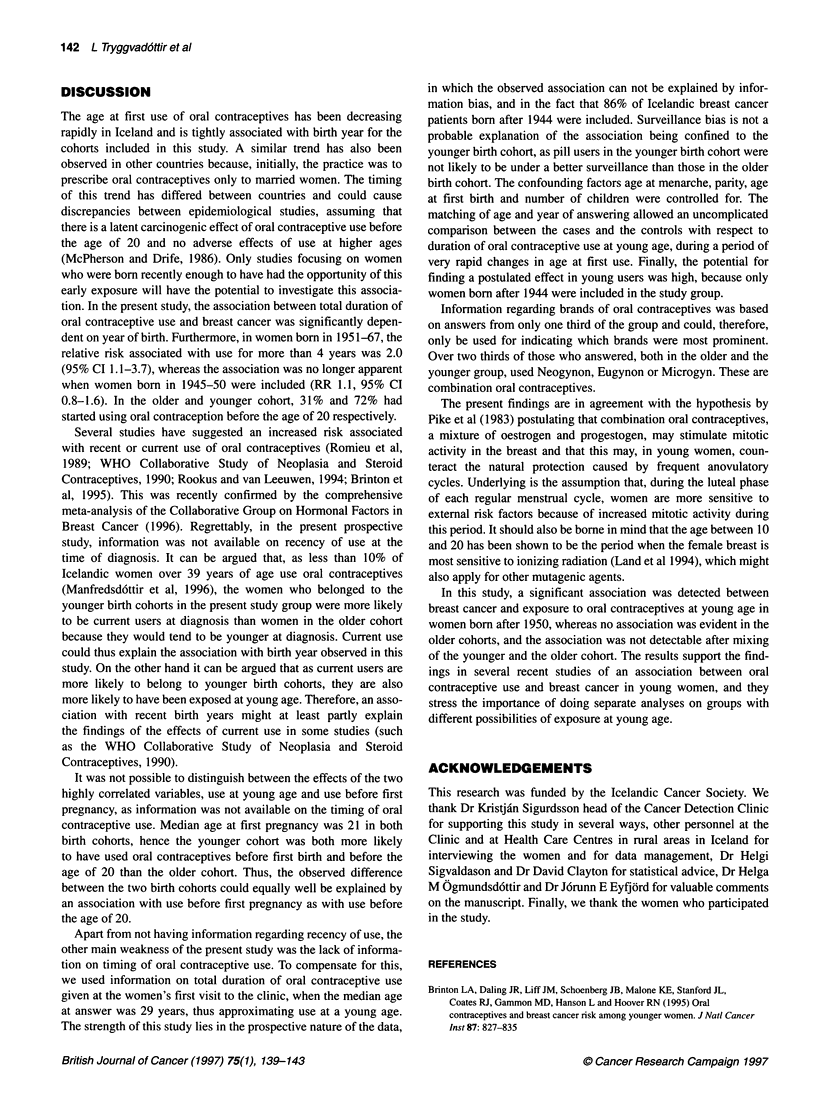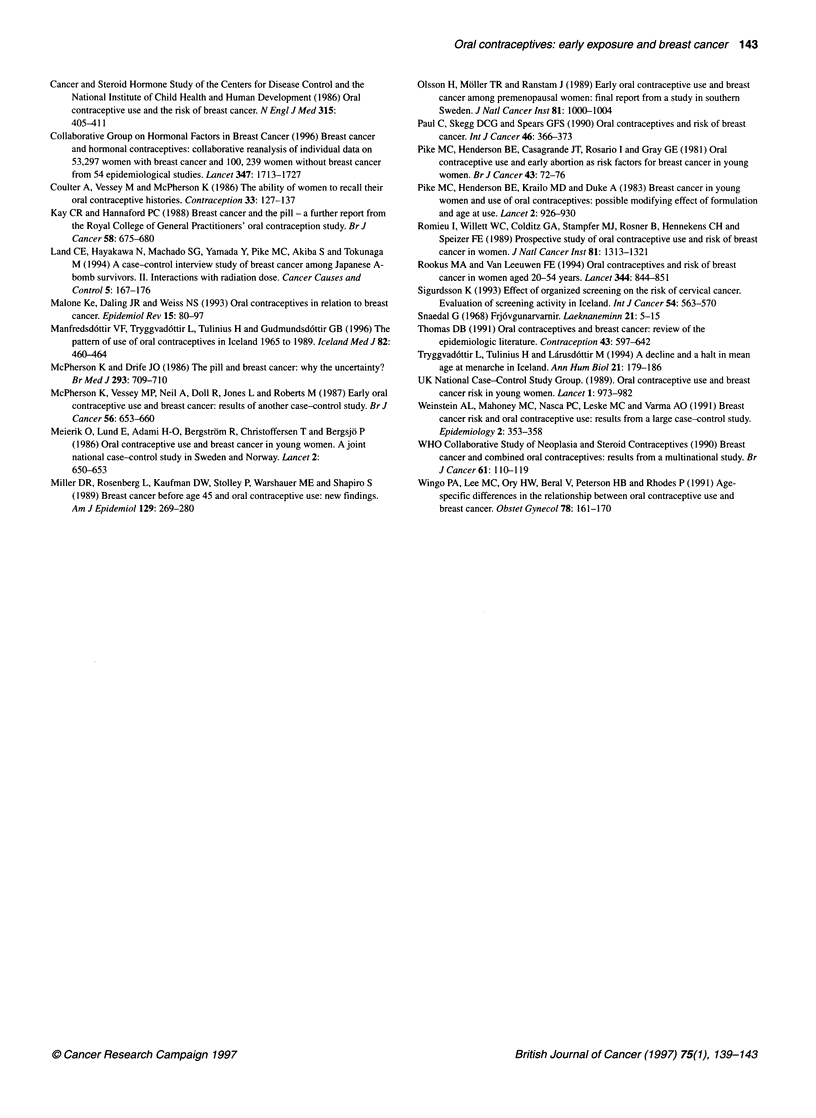# Oral contraceptive use at a young age and the risk of breast cancer: an Icelandic, population-based cohort study of the effect of birth year.

**DOI:** 10.1038/bjc.1997.23

**Published:** 1997

**Authors:** L. TryggvadÃ³ttir, H. Tulinius, G. B. GudmundsdÃ³ttir

**Affiliations:** Epidemiological Unit, Icelandic Cancer Society, Reykjavik.

## Abstract

The possible association between breast cancer and oral contraceptive use before the age of 20 was investigated using Icelandic population-based information from women born after 1944. The design was a nested case-control study within a cohort, using data on duration of oral contraceptive use at young ages. The availability of oral contraceptives before the age of 20 has changed dramatically and is highly dependent on birth years, with 20% and 82% starting before the age of 20 among Icelandic users born in 1945-47 and 1963-67 respectively. The association between total duration of oral contraceptive use and breast cancer was significantly dependent on year of birth. In women born in 1951-67 (based on 81 cases), the relative risk (RR) associated with use for more than 4 years was 2.0 (95% CI 1.1-3.7). The association disappeared when women born in 1945-50 were included (RR 1.1,95% CI 0.8-1.6), adding 123 cases. A significant trend of increased risk with longer duration was present only in the group born after 1950, with RR 0.9, 1.7 and 3.0 for < or = 4 years, >4-8 years and > 8 years of use respectively. The results of this study indicate an association between breast cancer and oral contraceptive use at a young age. They also stress the importance of distinguishing between groups with different opportunities for exposure at young age.


British Journal of Cancer (1997) 75(1), 139–143
© 1997 Cancer Research Campaign

### Oral contraceptive use at a young age and the risk of breast cancer: an Icelandic, population-based cohort study of the effect of birth year

**L Tryggvadóttir[1], H Tulinius[2,3] and GB Gudmundsdóttir[1]**

[1]Epidemiological Unit, Icelandic Cancer Society, PO Box 5420, IS-125 Reykjavik, Iceland; [2]Icelandic Cancer Registry, Icelandic Cancer Society, PO Box 5420, IS-125 Reykjavik, Iceland, [3]Department of Preventive Medicine, University of Iceland, Sudurgata, 101 Reykjavik, Iceland

**Summary** The possible association between breast cancer and oral contraceptive use before the age of 20 was investigated using Icelandic population-based information from women born after 1944. The design was a nested case–control study within a cohort, using data on duration of oral contraceptive use at young ages. The availability of oral contraceptives before the age of 20 has changed dramatically and is highly dependent on birth years, with 20% and 82% starting before the age of 20 among Icelandic users born in 1945–47 and 1963–67 respectively. The association between total duration of oral contraceptive use and breast cancer was significantly dependent on year of birth. In women born in 1951–67 (based on 81 cases), the relative risk (RR) associated with use for more than 4 years was 2.0 (95% CI 1.1–3.7). The association disappeared when women born in 1945–50 were included (RR 1.1, 95% CI 0.8–1.6), adding 123 cases. A significant trend of increased risk with longer duration was present only in the group born after 1950, with RR 0.9, 1.7 and 3.0 for ≤4 years, >4–8 years and > 8 years of use respectively. The results of this study indicate an association between breast cancer and oral contraceptive use at a young age. They also stress the importance of distinguishing between groups with different opportunities for exposure at young age.

**Keywords:** breast neoplasm; women; contraceptives; oral

Much of the research on the association between oral contraceptive use and breast cancer risk has given inconclusive results (Thomas, 1991; Malone et al, 1993). The paper by the Collaborative Group on Hormonal Factors in Breast Cancer (1996) was published as this paper went to press. Oral contraceptives were introduced around 1960 and were prescribed (initially) mainly for married women. In Britain, oral contraceptive use early in life became more common in the early 1970s and in the USA in the late 1970s (McPherson and Drife, 1986). Differences are to be expected among other countries, complicating research on the effects of exposure at a young age because the birth cohorts, exposed at a young age, have only recently entered the age at which the risk of breast cancer is high. An association between oral contraceptive use at a young age and breast cancer would not be detected in studies including a wide range of birth cohorts because such studies would include too many women for whom there was no possibility of early exposure.

In recent years, several studies have demonstrated an association between oral contraceptive use and breast cancer when focusing on women diagnosed under the age of 45 and/or on young users (Pike et al, 1981; Meierik et al, 1986; McPherson et al, 1987; Kay and Hannaford, 1988; Miller et al, 1989; Olsson et al, 1989; UK National Case–Control Study Group, 1989; WHO Collaborative Study of Neoplasia and Steroid Contraceptives, 1990; Weinstein et al, 1991; Wingo et al, 1991; Rookus and van Leeuwen, 1994; Brinton et al, 1995). This may indicate effects in young users because women diagnosed under the age of 45 in recent years tend to belong to the birth cohorts exposed at a young age. However, studies on risk associated with early age at first use have also given inconclusive results (Cancer and Steroid Hormone Study, 1986; Meierik et al, 1986; Olsson et al, 1989; UK National Case–Control Study Group, 1989; Paul et al, 1990; Weinstein et al, 1991).

In Iceland, prescription practices have changed markedly since 1960. The age at first use of oral contraceptives has been rapidly decreasing and now nearly all new users start well before the age of 20, whereas most women born before 1950 started after the age of 20 (Manfredsdóttir et al, 1996).

Here, we report a study of the effects of oral contraceptive use at young age on the risk of breast cancer in cohorts of Icelandic women who had the possibility of exposure at least from the age of 20. We also report the effects of focusing on birth cohorts with a successively lower age at exposure. The study population was Icelandic women born after 1944 who participated in a nationwide cancer detection programme of the Icelandic Cancer Society. The design was a nested case–control study within a cohort.

### METHODS

Two sources of information were used: the population-based Icelandic Cancer Registry and the databank of the Cancer Detection Clinic of the Icelandic Cancer Society. The former covers all cancer cases diagnosed since 1954 in the Icelandic population, totalling around 260 000. The latter contains information from around 90 000 women participating in the cancer detection activities of the Cancer Society, starting in 1964 (Sigurdsson, 1993; Tryggvadóttir et al, 1994). Record linkage uses the personal identification number of the Icelandic National Roster.

*Received 22 March 1996*
*Revised 15 July 1996A*
*Accepted 25 July 1996*

*Correspondence to:* L Tryggvadóttir

**Table 1** Reproductive and menstrual characteristics of cases and matched controls, as reported at the first visit to the Clinic, for the total study group (204 cases, 1183 controls). Median age at interview was 29 years (range 18–43 years)

| | Number (%) | | | |
|---|---|---|---|---|
| | Cases | Controls | RR[a] | P-value |
| Age at menarche (year) | | | | |
| <13 | 78 (38) | 367 (31) | 1.0 | – |
| 13 | 69 (34) | 420 (36) | 0.8 | 0.12 |
| 14+ | 57 (28) | 396 (33) | 0.7 | 0.05 |
| Parous | | | | |
| Yes | 175 (86) | 1023 (86) | 1.0 | – |
| No | 29 (14) | 160 (14) | 1.2 | 0.53 |
| Age at first childbirth | | | | |
| <20 | 55 (27) | 329 (28) | 1.0 | – |
| 20–29 | 113 (55) | 682 (58) | 1.0 | 0.99 |
| 30+ | 7 ( 3) | 12 ( 1) | 3.9 | 0.01 |
| Number of children | | | | |
| 1 | 47 (23) | 289 (24) | 1.0 | – |
| 2 | 81 (40) | 399 (34) | 1.3 | 0.18 |
| 3+ | 47 (23) | 335 (28) | 0.9 | 0.75 |
| Oral contraceptive use | | | | |
| Never | 42 (21) | 226 (19) | 1.0 | – |
| >0 to ≤4 years | 116 (57) | 703 (59) | 0.9 | 0.72 |
| >4 to ≤8 years | 36 (18) | 201 (17) | 1.0 | 0.90 |
| >8 years | 10 ( 5) | 53 ( 4) | 1.3 | 0.55 |

[a]All variables included simultaneously.

**Table 2** Relative risk of breast cancer for oral contraceptive use >4 years *vs* ≤ 4 years by birth years[a], successively excluding earlier years of birth

| Birth years | Number of cases/controls | RR | 95% CI | P-value |
|---|---|---|---|---|
| *Total study group* | | | | |
| 1945–67 | 204/1183 | 1.1 | (0.8–1.6) | 0.50 |
| *Remaining years of birth* | | | | |
| 1946–67 | 180/1039 | 1.2 | (0.8–1.8) | 0.25 |
| 1947–67 | 151/865 | 1.4 | (0.9–2.1) | 0.14 |
| 1948–67 | 131/761 | 1.4 | (0.9–2.3) | 0.13 |
| 1949–67 | 115/669 | 1.5 | (0.9–2.4) | 0.11 |
| 1950–67 | 97/567 | 1.7 | (1.0–3.0) | 0.06 |
| 1951–67 | 81/472 | 2.0 | (1.1–3.7) | 0.02 |
| 1952–67 | 63/372 | 1.9 | (0.9–3.9) | 0.08 |
| 1953–67 | 55/323 | 2.2 | (1.0–4.7) | 0.05 |

[a]Adjusted for age at menarche, parity (yes/no), number of children and age at first birth.

At the Cancer Detection Clinic, information on various reproductive factors has been gathered by interviewer-administered questionnaires. Around 95% of Icelandic women born after 1929 have contributed information, most of them on more than one occasion. Internal comparison between repeated answers indicates satisfactory reliability of the data (Tryggvadóttir et al, 1994). The questions have changed with time, however from the beginning women have provided information regarding age at menarche, number of children and age at first birth.

A question regarding duration of oral contraceptive use was added in 1975, but information on the age at use of oral contraceptives was not collected and is therefore not available for the women who participated in this study. However, for descriptive purposes, a special survey was conducted by the Cancer Detection Clinic in 1991 and 1992 on age at first use of oral contraceptives. This information was used in the present study to describe changes in age at first use with descending birth years. A question regarding brand of oral contraceptives was introduced in 1979, however, although the response rate for most of the other questions is around 96%, the response rate for this question is only around 72%, mainly because of problems with recollection. It has been shown elsewhere that recall of brands is not accurate (Coulter et al, 1986).

In this study, the cases were restricted to women born after 1944. Oral contraceptives were used by less than 2% of women of childbearing age in Iceland before 1964 (Snaedal, 1968), and our intention was to include only women with a possibility of use at around the age of 20. The group of cases included all Icelandic women diagnosed with invasive breast cancer before 1 July 1995 who were born after 1944 and who had given information in the Cancer Detection Clinic databank between 1975 and 1993, but before the diagnosis of breast cancer. Information given before 1975 could not be used because of lack of information regarding duration of oral contraceptive use. The controls were randomly drawn from the Cancer Detection Clinic databank, matched on birth year, and year of first attendance to the Clinic.

As information regarding age at use of oral contraceptives was not available, we focused on information given at first attendance to approximate use at young age, the intention being to investigate the particular contribution of oral contraceptive use at young age.

**Table 3** Association between duration of oral contraceptive use and breast cancer for two birth cohorts

| Birth cohort | Duration of use (years) | RR[a] | 95% CI | P-value |
|---|---|---|---|---|
| 1945–50 | 0 | 1 | | – |
| (123 cases and 711 controls) | ≤4 | 1.0 | (0.6–1.7) | 0.96 |
| | >4–8 | 0.8 | (0.4–1.6) | 0.52 |
| | >8 | 0.8 | (0.3–2.5) | 0.75 |
| 1951–67[b] | 0 | 1 | | – |
| (81 cases and 472 controls) | ≤4 | 0.9 | (0.4–1.7) | 0.69 |
| | >4–8 | 1.7 | (0.7–3.8) | 0.23 |
| | >8 | 3.0 | (0.8–11.0) | 0.11 |

[a]Adjusted for age at menarche, number of children and age at first birth. [b]Trend for RR significant ($P$=0.02) when duration of oral contraceptive use was included as a continuous variable.

© Cancer Research Campaign 1997

**Figure 1** Oral contraceptive users. The graph gives the cumulative proportion of women who had started using oral contraception before the given age for successive birth cohorts

**Table 4** Brands of oral contraceptives reported by the participants in the study

| | Cases (%) | Controls (%) |
|---|---|---|
| *Cohort born in 1945–50* | *n* = 24 | *n* = 166 |
| Neogynon[a] | 17 | 29 |
| Eugynon[b] | 38 | 30 |
| Microgyn[c] | 12 | 10 |
| Gynovlar[d] | 12 | – |
| Delpregnin[e] | – | 10 |
| Other brands[g] | 21 | 22 |
| *Cohort born in 1951–67* | *n* = 40 | *n* = 24 |
| Neogynon | 40 | 31 |
| Eugynon | 32 | 22 |
| Microgyn | 15 | 27 |
| Conlumin[f] | – | 5 |
| Other brands[g] | 12 | 14 |

[a]Levonorgestrelum INN 0.25 mg and Ethinylestradiolum INN 50µg.
[b]Norgestrelum INN 0.5 mg and Ethinylestradiolum INN 50µg.
[c]Levonorgestrelum INN 0.15 mg and Ethinylestradiolum INN 30 µg.
[d]Norethisteronum INN, acetat, 3 mg and Ethinylestradiolum INN 50 µg.
[e]Megestroli acetats NFN 5 mg and Mestranolum NFN 0.1 mg.
[f]Norethisteronum INN 1 mg and Mestranolum INN 50 µg. [g]Only brands used by at least 5% of the group are listed. *n* = number of answers.

Therefore, in the main analysis, status at first visit was used in the model, both with respect to information on total duration of oral contraceptive use and on confounding factors. For cases who had first attended before 1975, we used first attendance after 1974 and, for them, controls were drawn from women with a similar pattern of attendance.

For estimating the relative risk of breast cancer, conditional logistic regression was applied, taking into account age at menarche, parity (yes/no), age at first birth and number of children. To investigate the effects of birth year on the observed association, an interaction term was included in the model (a multiplication factor between the variables 'year of birth' and 'duration of oral contraceptive use'). To investigate the effects of age at diagnosis, a similar interaction term was also included for this variable.

### RESULTS

A total of 236 breast cancer cases born after 1944 had been diagnosed in Iceland at the end of July 1995. Of those, 95% were identified in the Detection Clinic databank, of which 204 cases (86% of total) remained after exclusion of women who had not answered the questions after 1974 and/or only after diagnosis of breast cancer. The median age at diagnosis was 40 years (range 27–49 years). As controls, 1183 women were selected, on average 5.8 per case (range 3–7).

The women in the study were aged between 18 and 43 years at first visit, with a median age of 29 years. The reproductive and menstrual characteristics for cases and controls at the time of first visit are shown in Table 1, along with the relative risk for breast cancer. Young age at menarche, nulliparity and high age at first birth were significantly associated with increased breast cancer risk in this group of young women.

For the whole study group, no association was observed between duration of oral contraceptive use before first visit and breast cancer. The effect of birth years on the observed association with use for more than 4 years is shown in Table 2. When successively eliminating older birth cohorts, the RR increased and for women born in 1951 and later a significant RR of 2.0 was observed ($P$=0.02). The interaction term between year of birth and duration of oral contraceptive use entered the model ($P$=0.04). Table 3 shows the effects of duration of oral contraceptive use when the birth cohorts 1945–50 and 1951–67 were studied separately. In the former group, no effect was seen whereas, in the latter group, a significant trend was present when the total duration of oral contraceptive use was included as a linear continuous variable. The median age at first attendance was 30 years and 26 years in the older and younger birth cohort respectively. The upper limit of age at diagnosis was 43 years or lower for women born after 1950 compared with 49 years for the total group. An interaction term including age at diagnosis did not enter the model ($P$=0.41).

Figure 1 shows that the age at onset of oral contraceptive use in Iceland has steadily been decreasing for successive birth cohorts. The numbers are based on 8393 answers to the temporary questions regarding age at first use from 1991–92. The percentage of ever users was 90% or similar for all the birth cohorts in Figure 1. The cumulative percentage of oral contraceptive users who started before the age of 20 years was 20% and 82% for women born in 1945–47 and 1963–67 respectively. Furthermore, it was 31% and 72% in the birth cohorts 1945–50 and 1951–67 respectively. Of the women in the older cohort, 2% had started oral contraception before the age of 17, whereas 22% had started before the age of 17 in the younger cohort.

We looked at the information given regarding brands of oral contraceptives separately for the group born before 1951 and for the group born in 1951–67. As information on brands had not been collected before 1979, it was not available for approximately 50% of the study group who had given answers in 1975–78. This was more pronounced in the group of women born before 1951, as they had attended somewhat earlier for the first time. Furthermore, only around 72% of those who were asked remembered what brand they had used. The information shown in Table 4 is thus based on only 34% of the study group. Neogynon, Eugynon and Microgyn were reported by 63% of the cases born before 1951 and by 69% of the controls born in the same years. The same brands were reported by 85% of the cases born after 1950 and by 79% of the controls.

© Cancer Research Campaign 1997 

### DISCUSSION

The age at first use of oral contraceptives has been decreasing rapidly in Iceland and is tightly associated with birth year for the cohorts included in this study. A similar trend has also been observed in other countries because, initially, the practice was to prescribe oral contraceptives only to married women. The timing of this trend has differed between countries and could cause discrepancies between epidemiological studies, assuming that there is a latent carcinogenic effect of oral contraceptive use before the age of 20 and no adverse effects of use at higher ages (McPherson and Drife, 1986). Only studies focusing on women who were born recently enough to have had the opportunity of this early exposure will have the potential to investigate this association. In the present study, the association between total duration of oral contraceptive use and breast cancer was significantly dependent on year of birth. Furthermore, in women born in 1951–67, the relative risk associated with use for more than 4 years was 2.0 (95% CI 1.1–3.7), whereas the association was no longer apparent when women born in 1945–50 were included (RR 1.1, 95% CI 0.8–1.6). In the older and younger cohort, 31% and 72% had started using oral contraception before the age of 20 respectively.

Several studies have suggested an increased risk associated with recent or current use of oral contraceptives (Romieu et al, 1989; WHO Collaborative Study of Neoplasia and Steroid Contraceptives, 1990; Rookus and van Leeuwen, 1994; Brinton et al, 1995). This was recently confirmed by the comprehensive meta-analysis of the Collaborative Group on Hormonal Factors in Breast Cancer (1996). Regrettably, in the present prospective study, information was not available on recency of use at the time of diagnosis. It can be argued that, as less than 10% of Icelandic women over 39 years of age use oral contraceptives (Manfredsdóttir et al, 1996), the women who belonged to the younger birth cohorts in the present study group were more likely to be current users at diagnosis than women in the older cohort because they would tend to be younger at diagnosis. Current use could thus explain the association with birth year observed in this study. On the other hand it can be argued that as current users are more likely to belong to younger birth cohorts, they are also more likely to have been exposed at young age. Therefore, an association with recent birth years might at least partly explain the findings of the effects of current use in some studies (such as the WHO Collaborative Study of Neoplasia and Steroid Contraceptives, 1990).

It was not possible to distinguish between the effects of the two highly correlated variables, use at young age and use before first pregnancy, as information was not available on the timing of oral contraceptive use. Median age at first pregnancy was 21 in both birth cohorts, hence the younger cohort was both more likely to have used oral contraceptives before first birth and before the age of 20 than the older cohort. Thus, the observed difference between the two birth cohorts could equally well be explained by an association with use before first pregnancy as with use before the age of 20.

Apart from not having information regarding recency of use, the other main weakness of the present study was the lack of information on timing of oral contraceptive use. To compensate for this, we used information on total duration of oral contraceptive use given at the women's first visit to the clinic, when the median age at answer was 29 years, thus approximating use at a young age. The strength of this study lies in the prospective nature of the data, in which the observed association can not be explained by information bias, and in the fact that 86% of Icelandic breast cancer patients born after 1944 were included. Surveillance bias is not a probable explanation of the association being confined to the younger birth cohort, as pill users in the younger birth cohort were not likely to be under a better surveillance than those in the older birth cohort. The confounding factors age at menarche, parity, age at first birth and number of children were controlled for. The matching of age and year of answering allowed an uncomplicated comparison between the cases and the controls with respect to duration of oral contraceptive use at young age, during a period of very rapid changes in age at first use. Finally, the potential for finding a postulated effect in young users was high, because only women born after 1944 were included in the study group.

Information regarding brands of oral contraceptives was based on answers from only one third of the group and could, therefore, only be used for indicating which brands were most prominent. Over two thirds of those who answered, both in the older and the younger group, used Neogynon, Eugynon or Microgyn. These are combination oral contraceptives.

The present findings are in agreement with the hypothesis by Pike et al (1983) postulating that combination oral contraceptives, a mixture of oestrogen and progestogen, may stimulate mitotic activity in the breast and that this may, in young women, counteract the natural protection caused by frequent anovulatory cycles. Underlying is the assumption that, during the luteal phase of each regular menstrual cycle, women are more sensitive to external risk factors because of increased mitotic activity during this period. It should also be borne in mind that the age between 10 and 20 has been shown to be the period when the female breast is most sensitive to ionizing radiation (Land et al 1994), which might also apply for other mutagenic agents.

In this study, a significant association was detected between breast cancer and exposure to oral contraceptives at young age in women born after 1950, whereas no association was evident in the older cohorts, and the association was not detectable after mixing of the younger and the older cohort. The results support the findings in several recent studies of an association between oral contraceptive use and breast cancer in young women, and they stress the importance of doing separate analyses on groups with different possibilities of exposure at young age.

### ACKNOWLEDGEMENTS

This research was funded by the Icelandic Cancer Society. We thank Dr Kristján Sigurdsson head of the Cancer Detection Clinic for supporting this study in several ways, other personnel at the Clinic and at Health Care Centres in rural areas in Iceland for interviewing the women and for data management, Dr Helgi Sigvaldason and Dr David Clayton for statistical advice, Dr Helga M Ögmundsdóttir and Dr Jórunn E Eyfjörd for valuable comments on the manuscript. Finally, we thank the women who participated in the study.

### REFERENCES

Brinton LA, Daling JR, Liff JM, Schoenberg JB, Malone KE, Stanford JL, Coates RJ, Gammon MD, Hanson L and Hoover RN (1995) Oral contraceptives and breast cancer risk among younger women. *J Natl Cancer Inst* **87**: 827–835

© Cancer Research Campaign 1997

Cancer and Steroid Hormone Study of the Centers for Disease Control and the National Institute of Child Health and Human Development (1986) Oral contraceptive use and the risk of breast cancer. *N Engl J Med* **315**: 405–411

Collaborative Group on Hormonal Factors in Breast Cancer (1996) Breast cancer and hormonal contraceptives: collaborative reanalysis of individual data on 53,297 women with breast cancer and 100, 239 women without breast cancer from 54 epidemiological studies. *Lancet* **347**: 1713–1727

Coulter A, Vessey M and McPherson K (1986) The ability of women to recall their oral contraceptive histories. *Contraception* **33**: 127–137

Kay CR and Hannaford PC (1988) Breast cancer and the pill – a further report from the Royal College of General Practitioners' oral contraception study. *Br J Cancer* **58**: 675–680

Land CE, Hayakawa N, Machado SG, Yamada Y, Pike MC, Akiba S and Tokunaga M (1994) A case–control interview study of breast cancer among Japanese A-bomb survivors. II. Interactions with radiation dose. *Cancer Causes and Control* **5**: 167–176

Malone Ke, Daling JR and Weiss NS (1993) Oral contraceptives in relation to breast cancer. *Epidemiol Rev* **15**: 80–97

Manfredsdóttir VF, Tryggvadóttir L, Tulinius H and Gudmundsdóttir GB (1996) The pattern of use of oral contraceptives in Iceland 1965 to 1989. *Iceland Med J* **82**: 460–464

McPherson K and Drife JO (1986) The pill and breast cancer: why the uncertainty? *Br Med J* **293**: 709–710

McPherson K, Vessey MP, Neil A, Doll R, Jones L and Roberts M (1987) Early oral contraceptive use and breast cancer: results of another case–control study. *Br J Cancer* **56**: 653–660

Meierik O, Lund E, Adami H-O, Bergström R, Christoffersen T and Bergsjö P (1986) Oral contraceptive use and breast cancer in young women. A joint national case–control study in Sweden and Norway. *Lancet* **2**: 650–653

Miller DR, Rosenberg L, Kaufman DW, Stolley P, Warshauer ME and Shapiro S (1989) Breast cancer before age 45 and oral contraceptive use: new findings. *Am J Epidemiol* **129**: 269–280

Olsson H, Möller TR and Ranstam J (1989) Early oral contraceptive use and breast cancer among premenopausal women: final report from a study in southern Sweden. *J Natl Cancer Inst* **81**: 1000–1004

Paul C, Skegg DCG and Spears GFS (1990) Oral contraceptives and risk of breast cancer. *Int J Cancer* **46**: 366–373

Pike MC, Henderson BE, Casagrande JT, Rosario I and Gray GE (1981) Oral contraceptive use and early abortion as risk factors for breast cancer in young women. *Br J Cancer* **43**: 72–76

Pike MC, Henderson BE, Krailo MD and Duke A (1983) Breast cancer in young women and use of oral contraceptives: possible modifying effect of formulation and age at use. *Lancet* **2**: 926–930

Romieu I, Willett WC, Colditz GA, Stampfer MJ, Rosner B, Hennekens CH and Speizer FE (1989) Prospective study of oral contraceptive use and risk of breast cancer in women. *J Natl Cancer Inst* **81**: 1313–1321

Rookus MA and Van Leeuwen FE (1994) Oral contraceptives and risk of breast cancer in women aged 20–54 years. *Lancet* **344**: 844–851

Sigurdsson K (1993) Effect of organized screening on the risk of cervical cancer. Evaluation of screening activity in Iceland. *Int J Cancer* **54**: 563–570

Snaedal G (1968) Frjóvgunarvarnir. *Laeknaneminn* **21**: 5–15

Thomas DB (1991) Oral contraceptives and breast cancer: review of the epidemiologic literature. *Contraception* **43**: 597–642

Tryggvadóttir L, Tulinius H and Lárusdóttir M (1994) A decline and a halt in mean age at menarche in Iceland. *Ann Hum Biol* **21**: 179–186

UK National Case–Control Study Group. (1989). Oral contraceptive use and breast cancer risk in young women. *Lancet* **1**: 973–982

Weinstein AL, Mahoney MC, Nasca PC, Leske MC and Varma AO (1991) Breast cancer risk and oral contraceptive use: results from a large case–control study. *Epidemiology* **2**: 353–358

WHO Collaborative Study of Neoplasia and Steroid Contraceptives (1990) Breast cancer and combined oral contraceptives: results from a multinational study. *Br J Cancer* **61**: 110–119

Wingo PA, Lee MC, Ory HW, Beral V, Peterson HB and Rhodes P (1991) Age-specific differences in the relationship between oral contraceptive use and breast cancer. *Obstet Gynecol* **78**: 161–170

© Cancer Research Campaign 1997